# Titanium Dioxide-Coated Zinc Oxide Nanorods as an Efficient Photoelectrode in Dye-Sensitized Solar Cells

**DOI:** 10.3390/nano10081598

**Published:** 2020-08-14

**Authors:** Qiang Zhang, Shengwen Hou, Chaoyang Li

**Affiliations:** 1 School of Systems Engineering, Kochi University of Technology, Kami, Kochi 782-8502, Japan; 216005z@gs.kochi-tech.ac.jp; 2 Center for Nanotechnology, Kochi University of Technology, Kami, Kochi 782-8502, Japan; 186003p@gs.kochi-tech.ac.jp

**Keywords:** titanium dioxide, zinc oxide, core–shell nanorods, mist chemical vapor deposition, dye-sensitized solar cell

## Abstract

Well-arrayed zinc oxide nanorods applied as photoelectrodes for dye-sensitized solar cells were synthesized on an aluminum-doped zinc oxide substrate by the multi-annealing method. In order to improve the chemical stability and surface-to-volume ratio of photoanodes in dye-sensitized solar cells, the synthesized zinc oxide nanorods were coated with pure anatase phase titanium dioxide film using a novel mist chemical vapor deposition method. The effects of the titanium dioxide film on the morphological, structural, optical, and photovoltaic properties of zinc oxide–titanium dioxide core–shell nanorods were investigated. It was found that the diameter and surface-to-volume ratio of zinc oxide nanorods were significantly increased by coating them with titanium dioxide thin film. The power conversion efficiency of dye-sensitized solar cells was improved from 1.31% to 2.68% by coating titanium dioxide film onto the surface of zinc oxide nanorods.

## 1. Introduction

Since Grätzel et al. developed the titanium dioxide (TiO_2_)-based dye-sensitized solar cell (DSSC) in 1991 [1], the DSSC has emerged as a promising photovoltaic device, due to its promising power conversion efficiency (PCE), low fabrication cost, and low toxicity [2,3,4,5]. Hitherto, it has been reported that TiO_2_-based DSSCs achieved a notable PCE of over 14% [6]. However, further improvements in PCE are difficult to achieve due to some disadvantages in current TiO_2_-based DSSCs, such as the low carrier transportation rate of TiO_2_ resulting from its low electron mobility, as well as the difficulty in fabricating TiO_2_ nanostructures with a large surface-to-volume ratio [7,8]. Recently, zinc oxide (ZnO) has been widely investigated in different types of solar cells [9,10,11]. As an alternative photoanode material of DSSCs, ZnO has attracted much attention because it exhibits a similar bandgap and electron injection process from excited dye molecules to TiO_2_ [12,13]. Moreover, the electron mobility of ZnO (200~1000 cm^2^/(V∙s)) is much higher than that of TiO_2_ (0.1~4 cm^2^/(V∙s)) [14], which will enhance electron transportation. Additionally, compared with TiO_2_, it is much easier to fabricate ZnO as various nanostructures to enlarge the surface-to-volume ratio [15]. Therefore, ZnO-based nanostructures and nanocomposites have much potential for application as a photoanode material to improve the PCE of DSSC.

However, the poor chemical stability of ZnO in the acidic dye solution and electrolyte solution of DSSCs has hampered its wider applicability as a photoanode material in DSSCs [16]. Additionally, defects easily form in ZnO, which increases the Zn^2+^/dye complex and the electron–hole recombination at the interface [17,18,19,20]. In order to overcome the shortcomings of ZnO-based photoanodes, one solution is to coat a chemically stable shell onto the surface of as-deposited ZnO. This core–shell structure can passivate ZnO’s surface to reduce the complex and form an energy barrier, thereby reducing the electron–hole recombination [21]. Among different ZnO-based nanocomposites, one of the most promising structures is ZnO–TiO_2_’s core–shell nanostructure. According to the literature [22,23,24,25], the PCE of ZnO photoanode-based DSSCs can be improved by about one to five times by replacing the ZnO photoanode with a corresponding ZnO–TiO_2_ core–shell nanostructure. It is reported that ZnO’s nanostructure could be coated with TiO_2_ thin film using the sol–gel method [26], solution method [27], and atomic layer deposition [28]. However, the difficulties that arise with the uniformity and also in controlling the thickness of the TiO_2_ layer are still unsolved.

**Figure 1:**
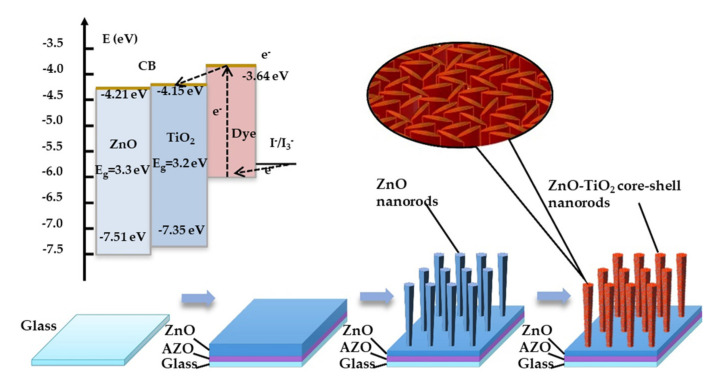
Fabrication mechanism and working principle of the zinc oxide–titanium dioxide (ZnO–TiO_2_) core–shell nanorod.

Based on our previous study, DSSCs with a high PCE could be achieved by controlling the vertical alignment of ZnO nanorods and the quality of transparent conductive substrates [29,30,31]. In addition, mist chemical vapor deposition (mist CVD) has been proven to be an effective method for modifying ZnO nanorods [32,33]. In this study, ZnO nanorods with vertical alignment were fabricated by a multi-annealing process in reducing ambient. Compared with ZnO nanorods fabricated by other methods, the ZnO nanorods fabricated by multi-annealing showed a higher concentration of oxygen vacancies. The oxygen vacancies were generated due to the effect of reducing ambient and they enhanced the conductivity of ZnO nanorods. However, the oxygen vacancies on the surface of ZnO nanorods will trigger the recombination of electrons. In order to solve this issue, the TiO_2_ thin layer was coated on ZnO nanorods by the mist CVD method to prevent the recombination of electrons and enhance the chemical stability of electrodes. Compared with other methods, the combination of the multiple annealing process and mist CVD method is an effective method to fabricate ZnO–TiO_2_ core–shell nanorods applied as photoelectrodes for DSSCs. Figure 1 shows the fabrication mechanism and working principle of ZnO–TiO_2_ core–shell nanorods. The electrons are injected from excited dye molecules to the conduction band (CB) of TiO_2_. Then, the electrons are transferred from the CB of TiO_2_ to the CB of ZnO. The ZnO core has high electron mobility and the TiO_2_ shell can protect the ZnO core from corrosion and suppress the recombination of electrons. After coating, the obtained ZnO–TiO_2_ core–shell nanorods, as well as the as-deposited ZnO nanorods, were used to fabricate DSSCs for comparison. The effects of TiO_2_ coating on the properties of ZnO–TiO_2_ core–shell nanorods were investigated in detail.

## 2. Materials and Methods

### 2.1. Deposition of Thin Films

The aluminum-doped ZnO (AZO, 300 nm) thin films were deposited on alkali-free glass sheets (Eagle XG, Corning Inc., Corning, NY, USA) using a conventional radio frequency (RF, 13.56 MHz) magnetron sputtering system with an AZO target (2 wt.% Al_2_O_3_). Following the deposition of AZO films, ZnO films with a 500 nm thickness were deposited on AZO by the same sputtering system with a ZnO target (5N). Table 1 shows the deposition conditions of the AZO film and ZnO film. Argon was selected as the working gas, the flow rate of which was maintained at 30 sccm. During the deposition, the working distance and temperature were set and maintained at 60 mm and 150 °C, respectively. The pressure and RF power for AZO film deposition were maintained at 1 Pa and 60 W. For the deposition of ZnO, the pressure and RF power were held at 7 Pa and 180 W.

### 2.2. Fabrication of ZnO Nanorods

After sputtering deposition, the fabricated ZnO films were treated using a multi-annealing process in a conventional annealing furnace. As shown in Table 2, the temperature was firstly kept at 300 °C for 2 h in a forming gas ambient (H_2_:N_2_ = 1.96%) to increase the density of zinc seeds on the surface. Then, the temperature was increased to 450 °C and kept at this level for 3 h for forming gas to produce the ZnO nanorods. Before the third forming gas annealing process, oxygen was introduced into the furnace for 40 min for surface oxidation to avoid an excessive reducing reaction. For safety considerations, nitrogen was introduced for 5 min between the forming gas and oxygen annealing processes.

### 2.3. Fabrication of ZnO–TiO_2_ Core–Shell Nanorods

Finally, TiO_2_ film was coated onto the surface of the fabricated ZnO nanorods by a mist CVD system. Table 3 shows the deposition condition of the TiO_2_ film. An ethanolic titanium tetraisopropoxide (TTIP, purity > 95.0%, Wako Pure Chemical Industries, Ltd., Osaka, Japan) solution with a concentration of 0.10 mol/L was prepared as the precursor solution. Mist droplets were generated from the precursor solution by ultrasonic atomization (2.4 MHz) and transferred to the reaction chamber by compressed air. The sample of as-deposited ZnO nanorods was placed in the reaction chamber and heated to 450 °C during the coating process.

### 2.4. Fabrication of DSSC

The obtained ZnO–TiO_2_ core–shell nanorods, as well as the as-deposited ZnO nanorods, were applied as photoanodes to fabricate DSSCs for comparison. N719 (Sigma Aldrich, St. Louis, MO, USA) was used as a dye sensitizer. The photoanodes were immersed in an ethanoic dye solution with a concentration of 5 × 10^−4^ mol/L for 12 h. A solution containing 0.10 mol/L lithium iodine and 0.05 mol/L iodine was used as the electrolyte. A platinum-coated indium-doped tin oxide film on glass was applied as the counter-electrode. Six samples of DSSCs were fabricated and investigated to confirm their reproducibility.

### 2.5. Characterization

The morphological properties of the AZO film, as-deposited ZnO nanorods, and ZnO–TiO_2_ core–shell nanorods were evaluated using field emission scanning electron microscopy (FE-SEM, JSM-7400F, JEOL, Tokyo, Japan) and transmission emission microscopy (TEM, JEM 2100F, JEOL, Tokyo, Japan). The structural properties of the AZO film were measured by X-ray diffraction (XRD, ATX-G, Rigaku, Tokyo, Japan). The structural properties of the as-deposited ZnO nanorods and ZnO–TiO_2_ core–shell nanorods were investigated by grazing incidence X-ray diffraction (GIXRD, ATX-G, Rigaku, Tokyo, Japan). The optical properties of the as-deposited ZnO nanorods and ZnO–TiO_2_ core–shell nanorods were obtained using a spectrophotometer (U-4100, Hitachi, Tokyo, Japan). The fabricated DSSCs were characterized using a solar simulator (PEC-L01, AM 1.5 G, 100 mW/cm^2^, Peccell Technologies Inc., Yokohama, Japan) and a source meter (Keithley 2400, Keithley Instruments Inc., Solon, OH, USA). All of the measurements were carried out at room temperature.

## 3. Results

**Figure 2:**
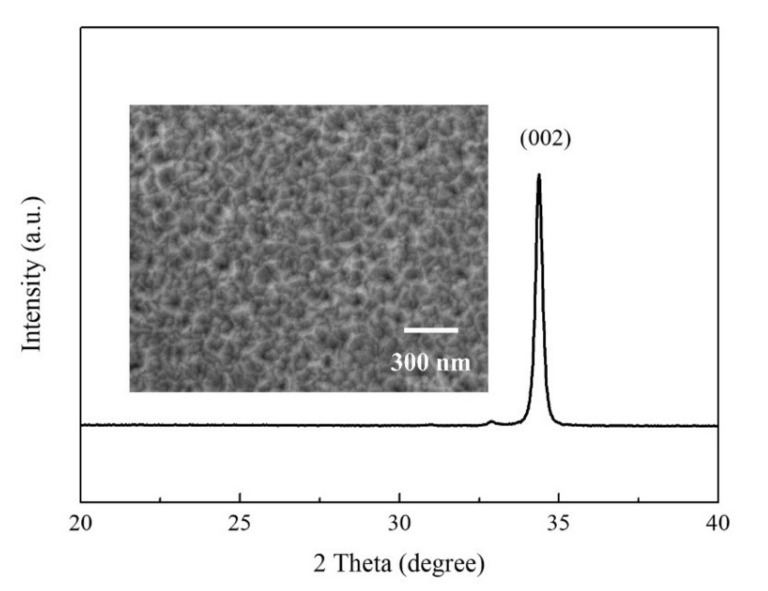
XRD pattern of AZO film (insert image shows the FE-SEM top view image of AZO film).

The XRD pattern of the AZO film is shown in Figure 2. It was found that only the (002) diffraction peak was observed in the XRD pattern, which indicated that the AZO films had highly (002) preferred orientation with a c-axis perpendicular to the substrates. The insert image in Figure 2 shows the FE-SEM top view image of the AZO film. It is confirmed that an AZO film with a uniform surface was obtained after deposition.

**Figure 3:**
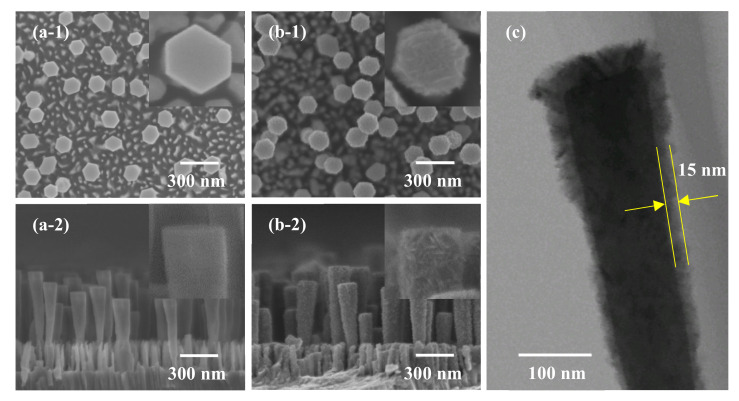
FE-SEM images of (**a**) as-deposited ZnO nanorods, (**b**) ZnO–TiO_2_ core–shell nanorods, and (**c**) TEM image of single ZnO–TiO_2_ core–shell nanorod ((**1**) Top view, (**2**) cross-section view).

The FE-SEM images of the as-deposited ZnO nanorods and ZnO–TiO_2_ core–shell nanorods are shown in Figure 3. The details of single nanorods are shown in the inset images. The as-deposited ZnO nanorods showed a well-arrayed hexagonal structure with a smooth surface. Compared with the as-deposited ZnO nanorods, the ZnO–TiO_2_ core–shell nanorods had a higher surface roughness and a larger diameter. Intertwined TiO_2_ nanosheets were observed on the surface of the ZnO–TiO_2_ core–shell nanorods, indicating that the TiO_2_ film was successfully coated onto the surface of the ZnO nanorods. Figure 3c shows the TEM image of a single ZnO–TiO_2_ core–shell nanorod. It was confirmed that the thickness of the TiO_2_ shell on the ZnO nanorods was around 15 nm.

**Figure 4:**
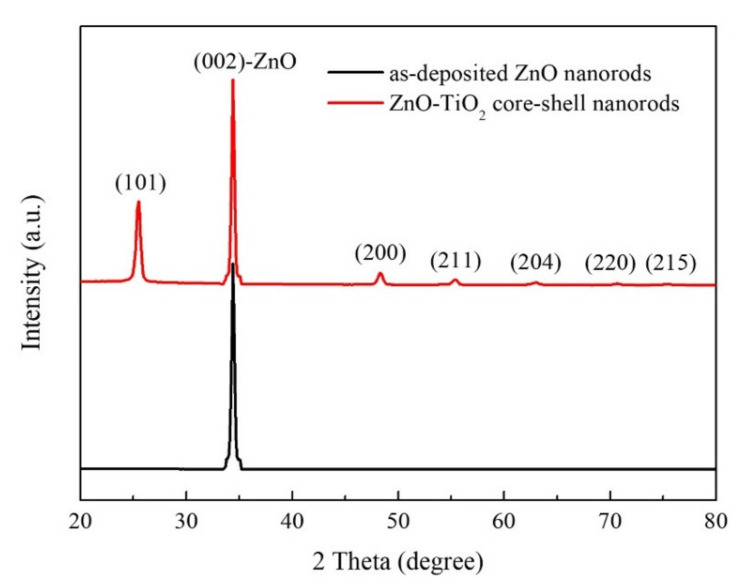
GIXRD patterns of as-deposited ZnO nanorods and ZnO–TiO_2_ core–shell nanorods.

The GIXRD patterns of the as-deposited ZnO nanorods and ZnO–TiO_2_ core–shell nanorods are shown in Figure 4. It was found that only the (002) diffraction peak was observed in the GIXRD pattern of the as-deposited ZnO nanorods, suggesting that both the ZnO film and ZnO nanorods had highly (002) preferred orientation with a c-axis perpendicular to the substrates. This agrees well with the FE-SEM results. In the GIXRD pattern of the ZnO–TiO_2_ core–shell nanorods, the observed peaks corresponded with the (101), (200), (211), (204), (220), and (215) diffraction peaks of the anatase phase TiO_2_ and the (002) diffraction peak of ZnO. All of the diffraction peaks of TiO_2_ were identified and corresponded with the anatase phase of TiO_2_ (JCPDS 21-1272), indicating that the TiO_2_ film coated on ZnO nanorods was pure anatase phase.

**Figure 5:**
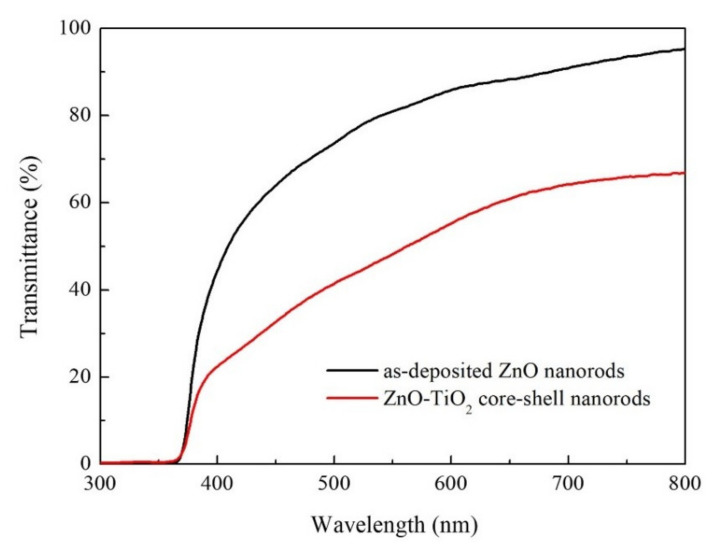
Optical transmission spectra of as-deposited ZnO nanorods and ZnO–TiO_2_ core–shell nanorods.

**Figure 6:**
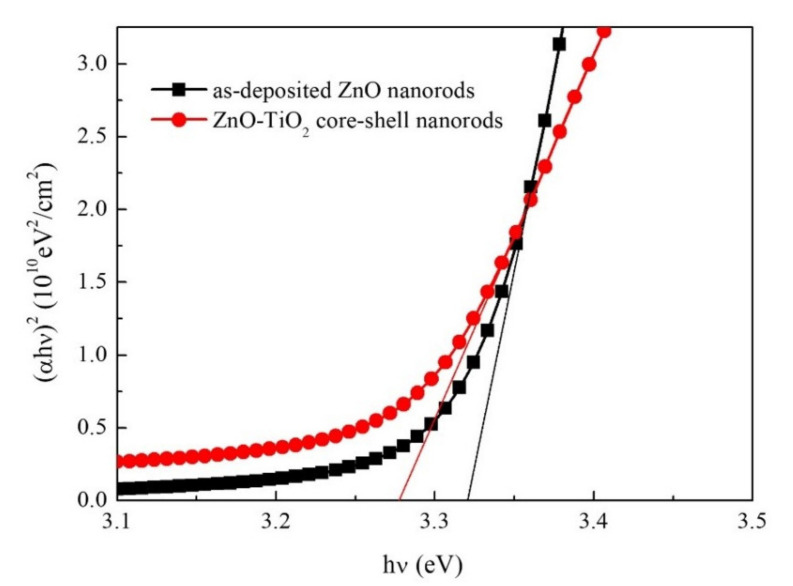
Variation of (*ahν*)^2^ of the as-deposited ZnO nanorods and ZnO–TiO_2_ core–shell nanorods as a function of the photon energy (*hν*).

The optical transmission spectra of the as-deposited ZnO nanorods and ZnO–TiO_2_ core–shell nanorods are shown in Figure 5. The as-deposited ZnO nanorods showed a high transmittance of 75% in visible range. After coating with TiO_2_ film, the transmittance of the nanorods in visible range decreased to 55%, due to the scattering of TiO_2_ nanosheets. It is well-known that the bandgap of material can be calculated from the transmittance data by the following equations [34,35]:

(1) $$\alpha =\frac{1}{d}\ln\left(\frac{1}{T}\right)$$

(2) $${\left(\alpha h\nu \right)}^{2}=A\left(h\nu -E_{g}\right)$$

where *α* is the absorption coefficient, *d* the thickness of material, *T* the transmittance, *hν* the incident photon energy, *A* a constant, and *E_g_* the bandgap. A plot of *(αhν)^2^* as a function of *hν* made to determine *E_g_* by linear fitting is shown in Figure 6. After fitting, the bandgap of the as-deposited ZnO nanorods was determined as around 3.32 eV, corresponding with the bandgap of bulk ZnO (3.37 eV). The bandgap of the ZnO–TiO_2_ core–shell nanorods was around 3.28 eV, corresponding with the bandgap of anatase phase TiO_2_ (3.2 eV).

**Figure 7:**
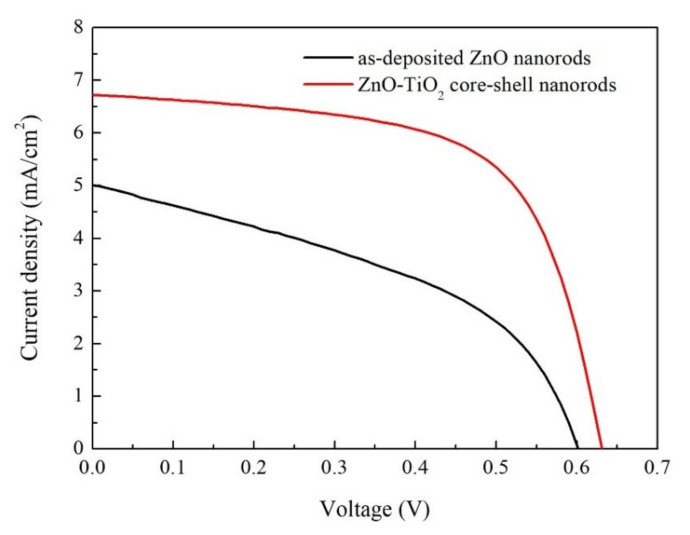
*J-V* characteristics of demonstrated dye-sensitized solar cells (DSSCs) applying as-deposited ZnO nanorods and ZnO–TiO_2_ core–shell nanorods as photoanodes.

Figure 7 shows the *J-V* characteristics of the demonstrated DSSCs applying as-deposited ZnO nanorods and ZnO–TiO_2_ core–shell nanorods as photoanodes. Compared with the DSSCs using as-deposited ZnO nanorods, the DSSCs applying ZnO–TiO_2_ core–shell nanorods showed higher open circuit voltage (*V_OC_*), higher short circuit current density (*J_SC_*), higher fill factor (*FF*), and higher PCE. After coating with TiO_2_, the *V_OC_* of the DSSCs increased from 0.60 V to 0.63 V, and the *J_SC_* increased from 5.01 mA/cm^2^ to 6.73 mA/cm^2^. It was found that the *FF* increased from 43.41% to 63.13%, and the PCE increased from 1.31% to 2.68%. The results showed good reproducibility by checking all of the DSSCs samples. The significant improvement of the *FF* and PCE was due to the great improvement in the *J_SC_*, which could be explained as follows: Firstly, the TiO_2_ shell increased the surface-to-volume ratio of the ZnO nanorods. Therefore, more dye molecules were absorbed onto the surface of the nanorods, which enhanced their light harvesting. Secondly, the TiO_2_ shell has a much lower electron–hole recombination rate than ZnO nanorods, which could greatly improve the efficiency of electron collection. Thirdly, the last step of a multi-annealing process was carried out in a reducing ambient. Consequently, many oxygen vacancies were generated on the surface of the ZnO nanorods. The oxygen vacancies acted as recombination centers, which triggered large amounts of recombination of electrons. After coating with TiO_2_ film, the recombination of electrons was suppressed. The efficient light harvesting and efficient electron collection contributed to the great improvement in the *J_SC_*.

## 4. Conclusions

Well-arrayed ZnO–TiO_2_ core–shell nanorods were successfully synthesized on AZO substrates by RF magnetron sputtering, multi-annealing, and the mist CVD method. The morphology of the ZnO nanorods was significantly changed by coating with a TiO_2_ film. After forming the ZnO–TiO_2_ core–shell structures, the diameter and surface-to-volume ratio of the nanorods were greatly increased. The PCE of DSSCs applying ZnO nanorods as photoanodes was increased two-fold from 1.31% to 2.68% by coating with TiO_2_.

## Figures and Tables

**Table 1:** Deposition conditions of AZO and ZnO films.

Film	AZO	ZnO
Target	AZO (2 wt.%)	ZnO (5N)
Working gas, Flow Rate (sccm)	Argon, 30	Argon, 30
Working distance (mm)	60	60
Deposition Temperature (°C)	150	150
Pressure (Pa)	1	7
RF Power (W)	60	180

**Table 2:** Annealing condition.

Step	Gas	Temperature (°C)	Time (min)
1	H_2_ in N_2_ (1.96%)	300	120
2	H_2_ in N_2_ (1.96%)	450	180
3	O_2_	450	40
4	H_2_ in N_2_ (1.96%)	450	120

**Table 3:** Deposition condition of TiO_2_.

**Solvent**	Ethanol
**Solute**	TTIP
**Concentration (mol/L)**	0.10
**Deposition Temperature (** **°C)**	450
**Carrier Gas, Flow Rate (L/min)**	Compressed Air (dried), 2.5
**Dilution Gas, Flow Rate (L/min)**	Compressed Air (dried), 4.5
